# Role of FBXW2 in explant cultures of bovine periosteum-derived cells

**DOI:** 10.1186/s13104-021-05825-z

**Published:** 2021-11-04

**Authors:** Mari Akiyama

**Affiliations:** Department of Biomaterials, Osaka Dental University, 8-1 Kuzuhahanozono-cho, Hirakata-shi, Osaka, 573-1121 Japan

**Keywords:** FBXW2, Osteocalcin, Periosteum, Periosteum-derived cells, Fluorescent immunostaining

## Abstract

**Objective:**

Bone regeneration is a potential technique for treating osteoporosis. A previous study reported that F-box and WD-40 domain-containing protein 2 (FBXW2) localized with osteocalcin in bovine periosteum after 5 weeks of explant culture. However, the osteoblastic functions of FBXW2 remain unclear. In this study, double-fluorescent immunostaining was used to investigate the potential role of FBXW2 and its relationship with osteocalcin.

**Results:**

At day 0, FBXW2 was expressed in the cambium layer between the bone and periosteum, while osteocalcin was expressed in bone. After explant culture, changes in the periosteum were observed from weeks 1 to 7. At week 1, partial FBXW2 expression was seen with a small amount of osteocalcin. At week 2, a layer of FBXW2 was observed. From weeks 3 to 7, tube-like structures of FBXW and osteocalcin were observed, and periosteum-derived cells were released from the periosteum in areas where no FBXW2 was observed. Bovine periosteum-derived cells can form a three-dimensional cell pellet, because multilayered cell sheets are formed inside of the periosteum in vitro. It is shown that in results FBXW2 is produced in periosteal explants near sites where initial osteogenic activity is observed, suggesting that it may be involved in periosteal osteogenesis.

**Supplementary Information:**

The online version contains supplementary material available at 10.1186/s13104-021-05825-z.

## Introduction

Osteoporosis and bone fracture can decrease the quality of life of patients. Bisphosphonates are used for the treatment of osteoporosis, but they are associated with the risk of osteonecrosis of the jaw [1]. As an alternative to bisphosphonates, bone regeneration is a potential treatment for osteoporosis. Many studies have investigated the role of the cambium layer of the periosteum in bone regeneration [2–5]; however, little is known about the specific proteins in the periosteum that aid in bone formation. Periosteal stem cells are also important for bone regeneration [6]. Bovine periosteum-derived cells are used for bone regeneration, and these cells can form multilayered cell sheets without scaffolds on tissue culture dishes [7]. To determine the mechanism of the formation of the multilayered cell sheets, the supernatant of cultured bovine and periosteum-derived cells and the periosteum have been studied [8, 9]. In a previous study, Akiyama investigated the supernatant of bovine periosteum-derived cells using mass spectrometry and immunohistochemistry [9], and found that F-box and WD-40 domain-containing protein 2 (FBXW2) was expressed in the periosteum [10]. FBXW2 is an F-box protein involved in the ubiquitin–proteasome system [11]. Among the 69 known F-box proteins, the functions of FBXW2, are unknown [12]. In 2018, Akiyama [13] reported that tube-like structures of FBXW2 localized with osteocalcin in the periosteum after 5 weeks of culture, but the relationship between these two proteins remains unclear. Akiyama [13] also reported that FBXW2 was expressed in the cambium layer located between the periosteum and bone; however, the osteoblastic function of FBXW2 is unknown. In this study, the periosteum and periosteum-derived cells were observed for up to 7 weeks using double-fluorescent immunostaining to determine the effects of FBXW2 on the osteoblastic characteristics of periosteum-derived cells.

## Main text

### Materials and methods

#### Animals

Japanese Black Cattle were raised in cattle farms for 30 months and slaughtered at the slaughterhouse for meat products (Kobe Chuo Chikusan, Kobe, Japan). Bovine legs were chosen by staff at the slaughterhouse, not by researchers. Explant culture started within 24 h after the animal’s death. This study used dead bovine legs, and no treatment or experimental intervention for living animals was performed.

#### Preparation of periosteal samples

The periosteum was separated from the bovine leg as described previously [7]. The observation period was from day 0 to 7 weeks. After 2 weeks of culture, without periosteum-derived cells, cell culture dishes and the periosteum were excluded. Explants that had contamination at any period were excluded. Legs from six different cows were used, and in some cases, the same cow at different time points was used. Bone sections were removed, fixed with 4% paraformaldehyde, and cast into paraffin blocks. The periosteum was cultured in 100-mm dishes in Medium 199 supplemented with 10% fetal bovine serum, 100 units/mL penicillin, and 100 μg/mL streptomycin (Wako Pure Chemical Industries, Ltd., Osaka, Japan), and 5 mg/mL ascorbic acid for up to 7 weeks. The medium was changed once a week. Every week, the periosteum was fixed and sections were prepared. Additional file 1: Fig. S1a, b shows the schema of this study. After the periosteum was peeled, cortical bone was excised and fixed. Before explant culture (day 0), the periosteum and bone with the cambium layer were investigated (Additional file 1: Fig. S1a). After explant culture, the periosteum and periosteum-derived cells were investigated at weeks 1 to 7 (Additional file 1: Fig. S1b).

**Fig. 1:**
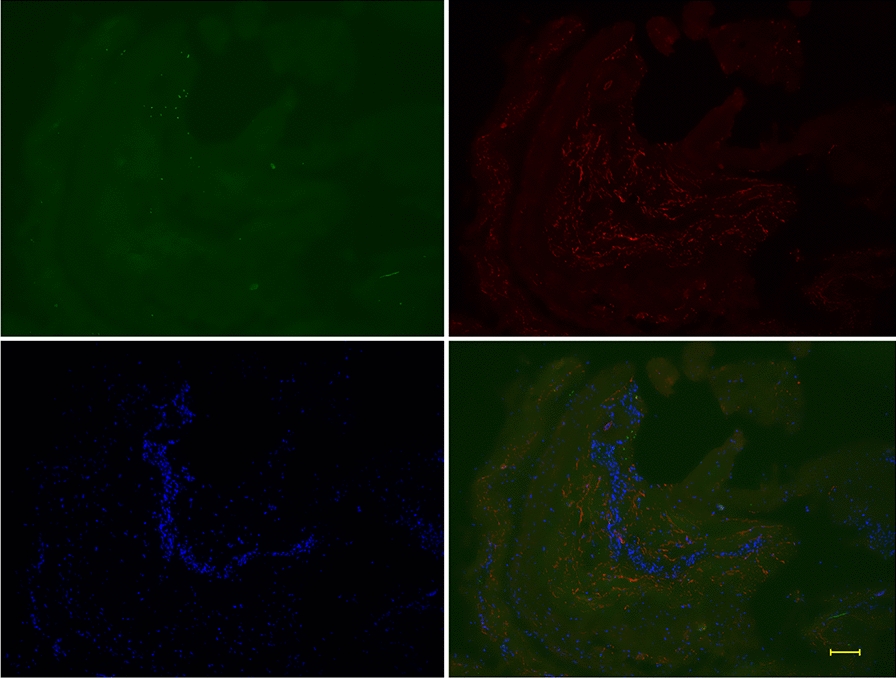
Double-fluorescent immunostaining of periosteum at day 0. FBXW2 is scarcely expressed in the periosteum, except in the cambium layer. Osteocalcin was not expressed. Osteocalcin: green; FBXW2: red; DAPI: blue. Scale bar: 100 μm

#### Fluorescent immunostaining and immunohistochemistry

All paraffin sections were pretreated with ready-to-use Proteinase K (Dako Cytomation, Glostrup, Denmark) for 10 min for antigen retrieval. The primary antibodies used were mouse anti-bovine osteocalcin monoclonal antibody (code no. M042, clone no. OCG2; Takara Bio Inc., Shiga, Japan) and goat anti-FBXW2 polyclonal antibody (#PA5-18,189; Invitrogen, Eugene, OR, USA). The secondary antibodies used were goat anti-mouse Alexa Fluor™ 488-labeled antibody (#A11029; Invitrogen), mouse anti-goat IgG-CFL 594 (no. sc516243; Santa Cruz Biotechnology, Inc., Dallas, TX, USA), and N-Histofine Simple Stain AP (multi; #414,261; Nichirei Biosciences Inc., Tokyo, Japan). The alkaline phosphatase-tagged antibodies were visualized with PermaRed/AP (K049; Diagnostic BioSystems, Pleasanton, CA, USA). Incubation with the anti-bovine osteocalcin monoclonal antibody (diluted 1:500) and anti-FBXW2 antibody (diluted 1:100) was performed for 4 h at room temperature. Cell nuclei were stained with hematoxylin or 4′,6-diamidino-2-phenylindole (DAPI). For the negative controls, an antibody against the receptor activator of NF-κB ligand (RANKL; No. sc-377079; Santa Cruz Biotechnology, Inc.) and normal goat serum (143–06,561; Fujifilm Wako Pure Chemical Industries, Ltd., Kanagawa, Japan) were used. Images were obtained using a fluorescence microscope (BZ-9000; Keyence Japan, Osaka, Japan) and BZ-II Viewer (Version1.1; Keyence) and BZ-II Analyzer (Version1.1; Keyence) software. One researcher performed all stages of the experiments and data analysis.

## Results

### Before explant culture (at day 0)

FBXW2 was expressed in the cambium layer of the periosteum, but showed little expression in bone (Additional file 2: Fig. S2a, b). Immunohistochemistry using alkaline phosphatase-labeled antibodies showed that osteocalcin was expressed around the lacuna of bone and at the border of the bone and periosteum (Additional file 2: Fig. S2c, d). Fluorescent immunostaining also showed that osteocalcin was expressed in bone (Additional file 2: Fig. S2e, f). At day 0, FBXW2 expression was scarcely detected in the periosteum, while FBXW2 expression was observed in the cambium layer and bone (Fig. 1). When the periosteum was excised from the bovine leg, the cambium layer containing FBXW2 was removed from the periosteum, and FBXW2 was therefore scarcely detected in the periosteum. Figure 1 also shows that osteocalcin was not expressed at day 0.

### Relationship between FBXW2 and osteocalcin

**Fig. 2:**
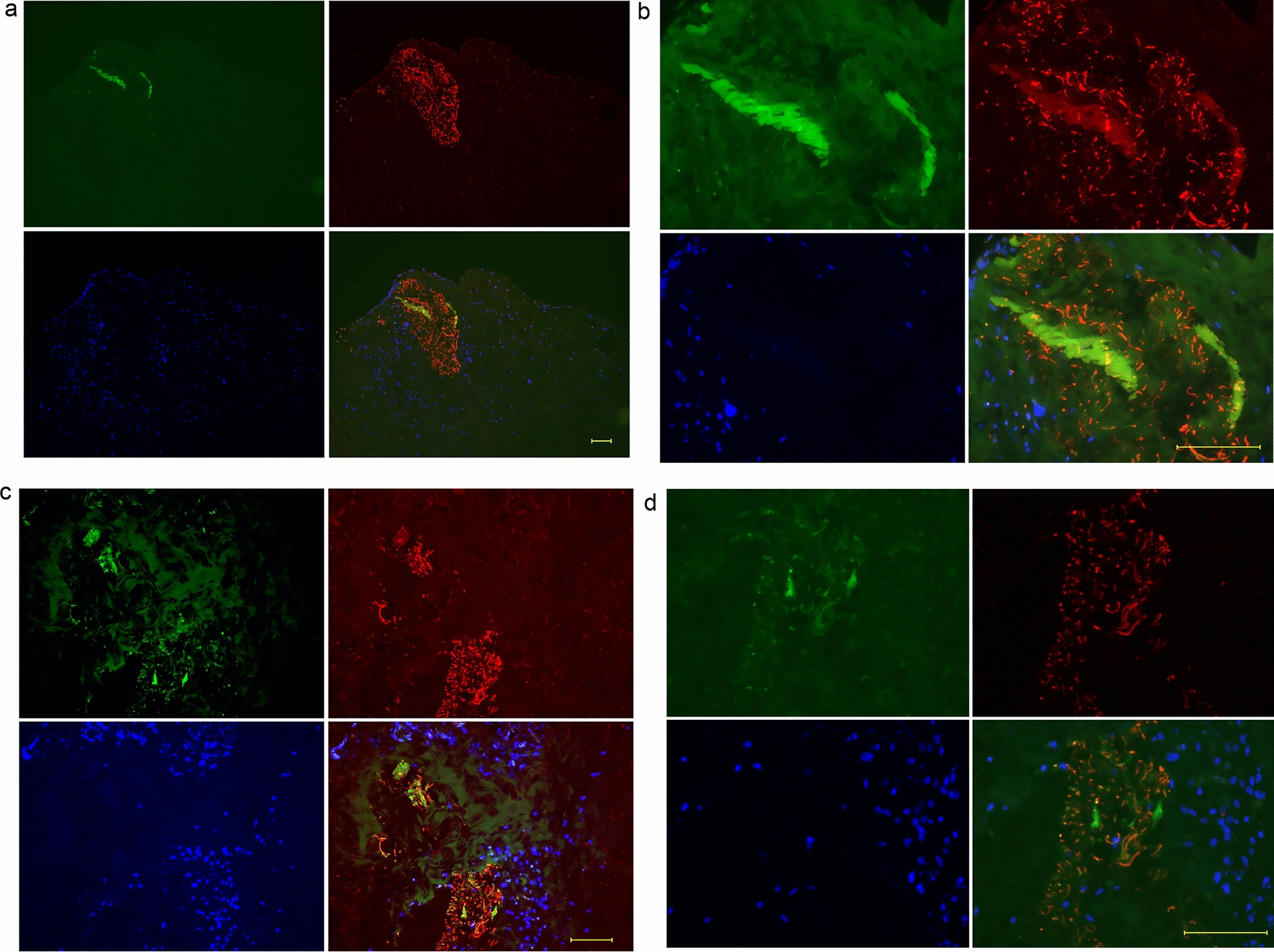

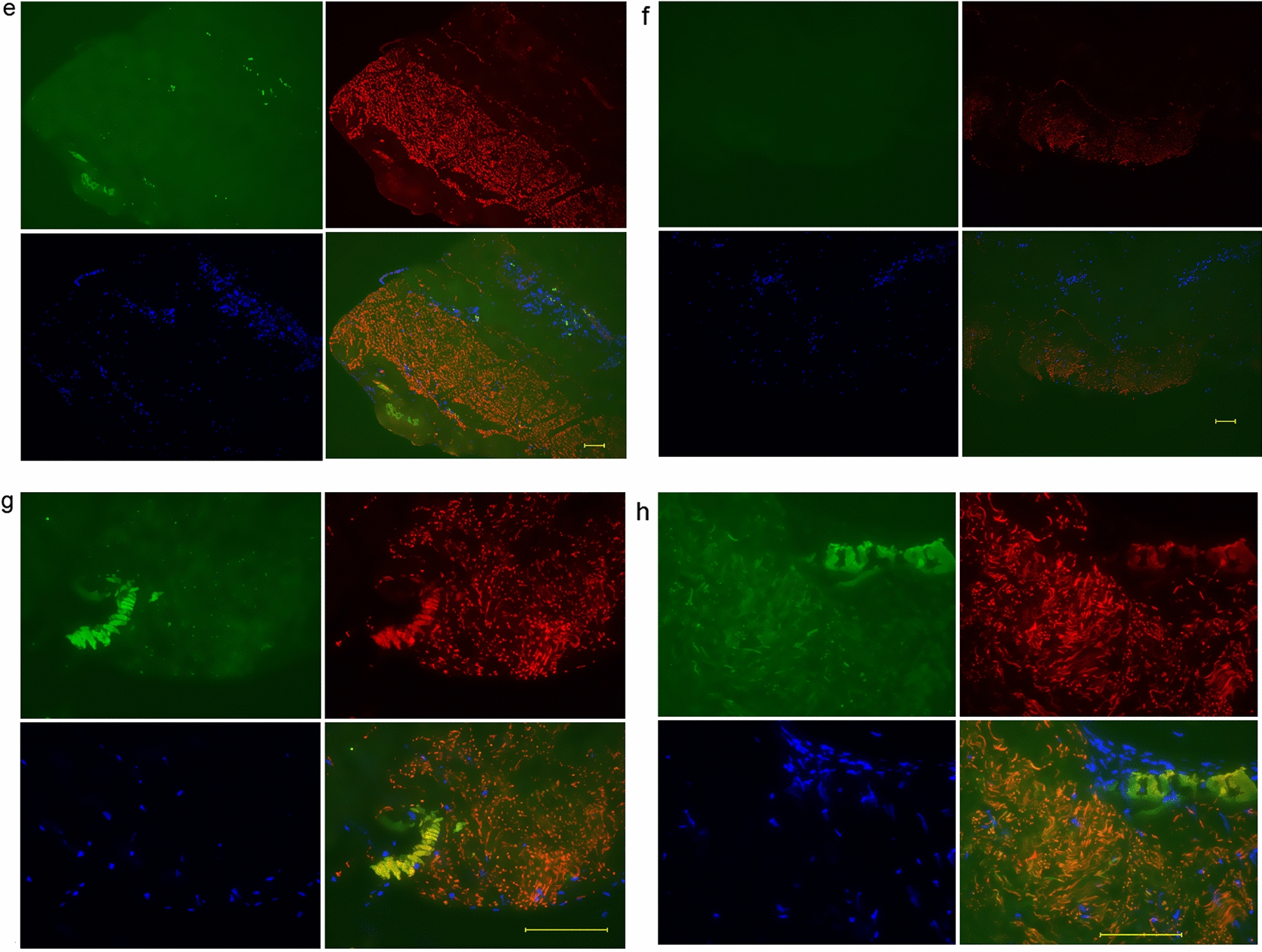
Double-fluorescent immunostaining of periosteum at weeks 1 and 2. At week 1, a small amount of osteocalcin appeared on FBXW2. A mass of osteocalcin appeared at week 2, when both osteocalcin and FBXW2 formed tube-like structures. **a–d** The periosteum at week 1. **b** A high-magnification image of (**a**), and **d** shows a high-magnification of **c**. **e**–**h** The periosteum at week 2. **f** RANKL: negative control for **e**. Scale bar: 100 μm. Osteocalcin: green; FBXW2: red; DAPI: blue

Figure 2 shows the relationship between FBXW2 and osteocalcin at weeks 1 (Fig. 2a–d) and 2 (Fig. 2e–h). Sections containing small amounts of osteocalcin with FBXW2 aggregation were observed at week 1 (Fig. 2a–d). Figure 2e shows a mass of osteocalcin that appeared, and that a thick layer of FBXW2 contacted a layer of osteocalcin. At 1–2 weeks, osteocalcin is found in the explants suggesting osteogenic activity. Figure 2f, which shows the negative control for Fig. 2e, shows that RANKL was not expressed. Figure 2g, h shows the mass of osteocalcin at week 2 and FBXW2 seems to be produced in the same regions, with FBXW2 showing a fiber-like structure under higher magnification.

### Potential role of FBXW2

**Fig. 3:**
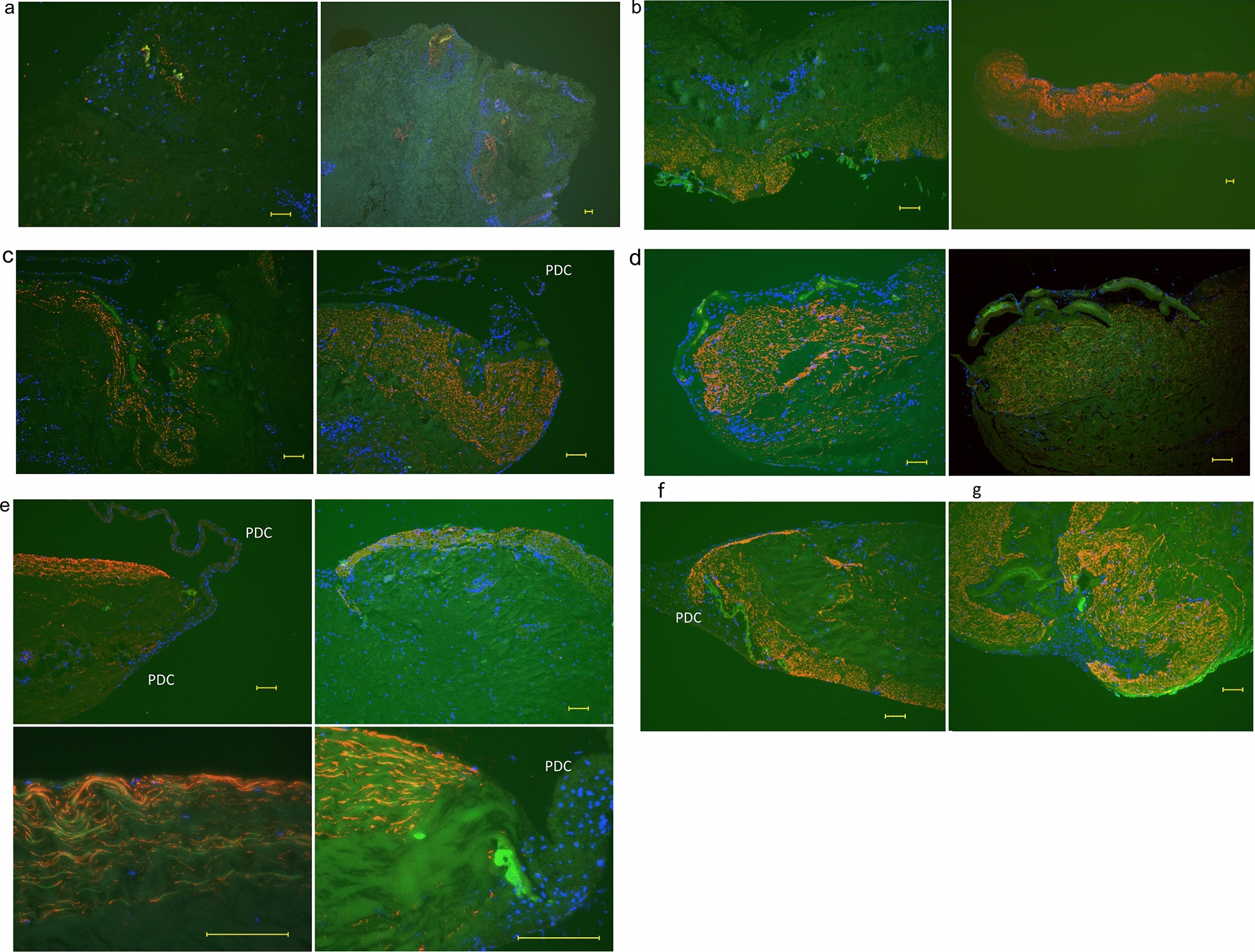
Double-fluorescent immunostaining of periosteum from weeks 1 to 7. **a** The periosteum at week 1. **b** The periosteum at week 2. **c** The periosteum at week 3. **d** The periosteum at week 4. **e** The periosteum at week 5. **f** The periosteum at week 6. **g** The periosteum at week 7. Scale bar: 100 μm. Osteocalcin: green; FBXW2: red; DAPI: blue. Red arrows indicate the layer of FBXW2. PDC: periosteum-derived cells

The relationship between FBXW2 and periosteum-derived cells with osteocalcin in the periosteum from weeks 1 to 7 is shown in Fig. 3a–g. In Fig. 3a, FBXW2 was partially expressed, and a small amount of osteocalcin was expressed near the FBXW2. At week 2, FBXW2 formed a layer, and osteocalcin was detected above the FBXW2 layer (Fig. 3b). At week 3, the periosteum-derived cells were distinct (Fig. 3c). At 3–7 weeks, the amounts of osteocalcin and FBXW2 increase, FBXW2 is found in a thick layer of tissue and there seems to be a separation between the regions where osteocalcin are found and those where FBXW2 is found. At sites where periosteum-derived cells are growing out of the explants, no FBXW2 is seen (Fig. 3c–g). Additional file 3: Figure S3 shows the periosteum at day 3 with periosteal cell synthesis of FBXW2 (Additional file 3: Fig. S3a) and tube-like structures of FBXW2 bursting out of the cells (Additional file 3: Fig. S3b).

## Discussion

Additional file 4: Table S1 shows the changes in FBXW2 and osteocalcin expression. Simon et al. [14] reported that surgical stimulation of the periosteum by a sharp incision caused cambium cell proliferation and new bone formation. They concluded that the cause of the new bone formation was an increase in the thickness of the cambium layer. In this study, before explant culture, the FBXW2 in the cambium layer was always in contact with osteocalcin in bone. At the beginning of explant culture, the periosteum was peeled from bone and placed on culture dishes without osteocalcin. FBXW2 might participate in osteocalcin synthesis as a result of such stimulation. It is also possible that FBXW2 might be used to monitor the amount of osteocalcin in bone.

In a previous study, at 5 weeks, both FBXW2 and osteocalcin in the periosteum formed thin tube-like structures similar to the structures observed at week 2 in this study (Fig. 2g, h), and FBXW2 was coated with osteocalcin [13]. Figure 2g, h shows the accumulation of osteocalcin and the tube-like structures of FBXW2 and osteocalcin. The start point region of periosteum-derived cells with osteocalcin included no FBXW2 (Fig. 3e). A possible reason for this finding is that FBXW2 might not have been synthesized in these regions. Another reason might be that FBXW2 and osteocalcin were expressed in the same region, and then FBXW2 was degraded and osteocalcin remained (see Fig. 2b, g, h). Tube-like structures of FBXW2 and osteocalcin were observed (Fig. 3e). Part of the tube-like structures of FBXW2 were degraded. As such, FBXW2 may gather osteocalcin around itself before being degraded, indicating that FBXW2 might function in condensing osteocalcin. Furthermore, the layer of FBXW2 with high cellularity did not cause outgrowth of periosteal cells (Fig. 3e) and the multilayered cell sheets formed inside the periosteum (Fig. 3d). FBXW2 might function in anchoring the multilayered cell sheets inside the periosteum.

In 2015, Hirashima et al. [15] revealed the anchoring structure of the calvarial periosteum. However, the components of the anchoring structure remain unclear. Sun’s group [16, 17] reported that FBXW2 suppressed lung cancer cell migration and invasion by inhibiting the escape of the cells, and in their working model, FBXW2 was expressed in the cytoplasm. However, in 2018, Akiyama [13] reported the expression of FBXW2 in the periosteum as an extracellular protein. Whereas Sun’s group investigated intracellular FBXW2 in relation to ubiquitination and degradation, the present study showed the periosteal cell synthesis of FBXW2 (Additional file 3: Fig. S3a) as well as tube-like structures of FBXW2 bursting out of cells (Additional file 3: Fig. S3b).

The alkaline phosphatase-labeled antibody against osteocalcin that was used in the present study was the same as that used in a previous study [13]; regardless of exposure time and autofluorescence, non-specific reactions were not observed, except for blood cells (data not shown). As shown in Fig. 2f, the anti-RANKL antibody was used as a negative control under the same conditions (mouse IgG_1_ concentration and treatment time of 4 μg/mL and 4 h, respectively).

The periosteum consists of a cambium layer and a fibrous layer in vivo. The cambium layer is osteogenic and the fibrous layer is poorly osteogenic. In this study, FBXW2 and osteocalcin were in contact with each other in vivo (Additional file 4: Table S1, Additional file 2: Fig. S2a, e). FBXW2 is located in the cambium layer, while osteocalcin is not located in either layer. At day 0 when preparing periosteal explants, part of the cambium layer and FBXW2 might have been peeled off with the fibrous layer. An explanation for this possibility is that a small amount of osteocalcin derived from the cambium layer remained in the explants. However, the cambium layer in vivo is thin. At day 0 in this study, FBXW2 expression in the explants was scarce (Additional file 4: Table S1, Fig. 1). After 2 weeks of explant culture, the FBXW2 layer was thick and obvious (Fig. 2e). With a thick layer of FBXW2, osteocalcin also become obvious. FBXW2 staining was found in some regions of osteocalcin staining, but osteocalcin staining was sometimes found where no FBXW2 was observed. The reason for this finding might be a difference in the cutting angle of sections and different times of cutting. Both in vivo and in vitro FBXW2 were in contact with osteocalcin. The reason for this being so is unclear. One possible reason is that FBXW2 re-formed the cambium layer in the explants. Periosteum-derived cells and their osteogenic ability might be caused by proteins other than FBXW2. Future studies need to investigate the association between FBXW2 and osteogenicity by knockdown of FBXW2.

## Limitations

Legs from six different cows were used, and in some cases, the same cow at different time points was used. With regard to the reproducibility of the results, differing results were sometimes obtained from the same sample owing to differences in the region of the section observed or the expression time. In particular, osteocalcin was expressed after week 1 in some cases. Although the reproducibility was not perfect, by using six different cows from weeks 1 to 7, this study showed a trend in the changes at each time point, which provided an approximate guideline for changes over time. The role of FBXW2 in this study is purely speculative without mechanistic experiments (e.g., knockdown of FBXW2).

## Supplementary Information

**Additional file 1: Fig. S1.** Schema of this study. **a** At day 0, the periosteum, cambium layer, and bone were observed. **b** From weeks 1 to 7, changes in the periosteum were observed in explant cultures.

**Additional file 2: Fig. S2.** Fluorescent immunostaining and immunohistochemistry of bone at day 0. FBXW2 is expressed in the cambium layer, and osteocalcin is expressed in bone. Scale bar: 100 μm. (a) FBXW2: red. (b) Negative control for (a). (c) Osteocalcin. (d) RANKL: negative control for (c). (e) Osteocalcin: green. (f) RANKL: negative control for (e). P: periosteum; B: bone; LOB: lacuna of bone.

**Additional file 3: Fig. S3.** Synthesis of FBXW2. Double-fluorescent immunostaining of the periosteum at day 3 showing periosteal cell synthesis of FBXW2 and tube-like structures of FBXW2 bursting out of the cells. Scale bar: 100 μm. Osteocalcin: green; FBXW2: red; DAPI: blue.

**Additional file 4: Table S1.** Changes in the expression of FBXW2 and osteocalcin.

## Data Availability

A pre-print version of this article is available on bioRxiv (https://www.biorxiv.org/content/10.1101/2020.12.17.423216v1). The content of this article has not been published previously and is not under consideration for publication elsewhere. Bovine legs were obtained from Kobe Chuo Chikusan (Kobe, Japan). All primary antibodies and secondary antibodies are commercially available. Accession number of FBXW2_BOVIN is Q58D00 or Q58DP3 (Database: SwissProt).

## Abbreviations

FBXW2: F-box and WD-40 domain-containing protein 2
RANKL: Receptor activator of NF-κB ligand
DAPI: 4′,6-Diamidino-2-phenylindole
