# Supplementary figures and images for: Role of FBXW2 in explant cultures of bovine periosteum-derived cells
Source: BMC Res Notes. 2021 Nov 4;14:410. doi: 10.1186/s13104-021-05825-z (PMC8569954; doi:10.1186/s13104-021-05825-z)

## Slide 1
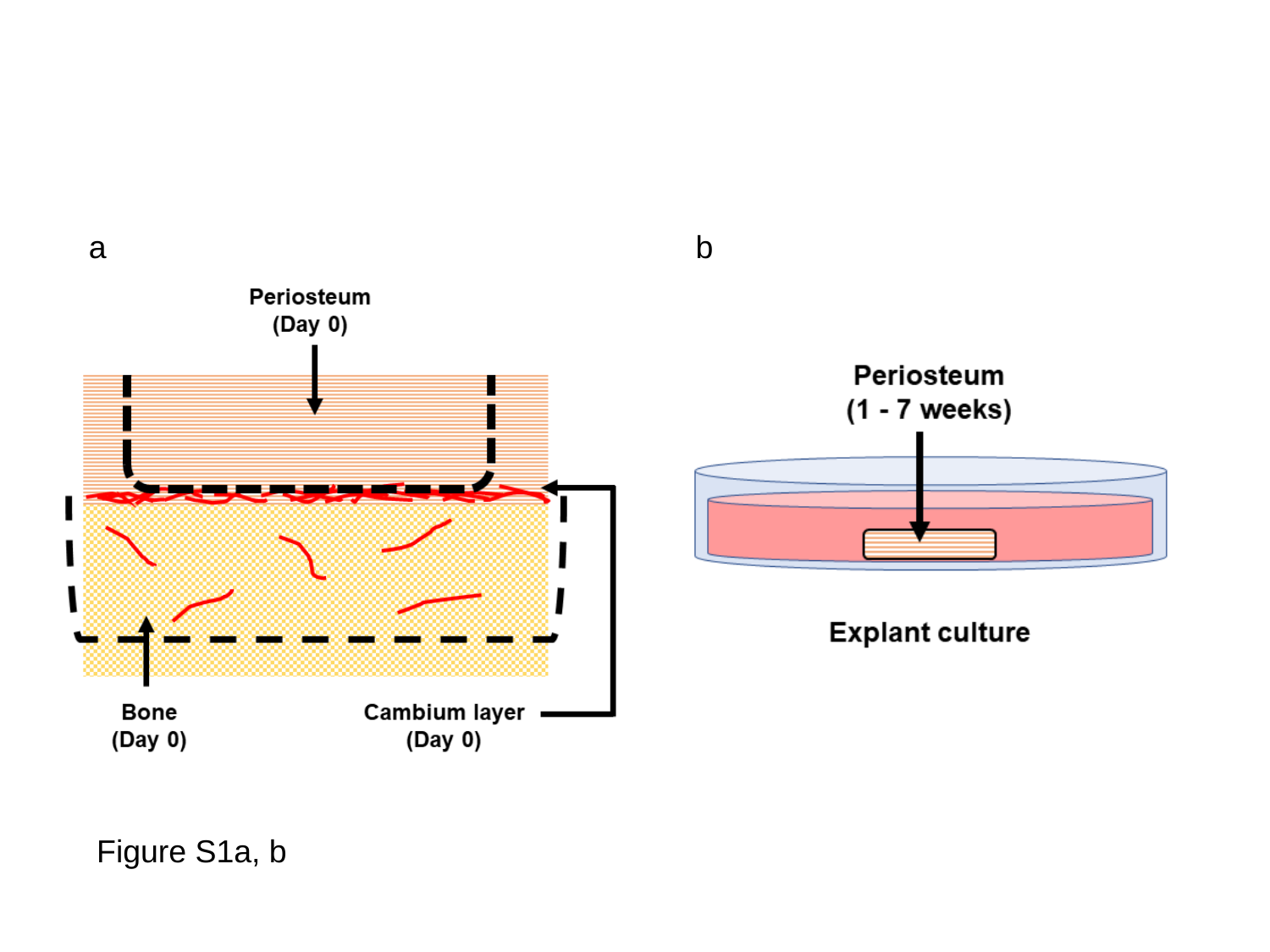

a
b
Figure S1a, b

Supplement: Supplementary file 1 — Additional file 1: Fig. S1. Schema of this study. a At day 0, the periosteum, cambium layer, and bone were observed. b From weeks 1 to 7, changes in the periosteum were observed in explant cultures. [file 13104_2021_5825_MOESM1_ESM.pptx]

a

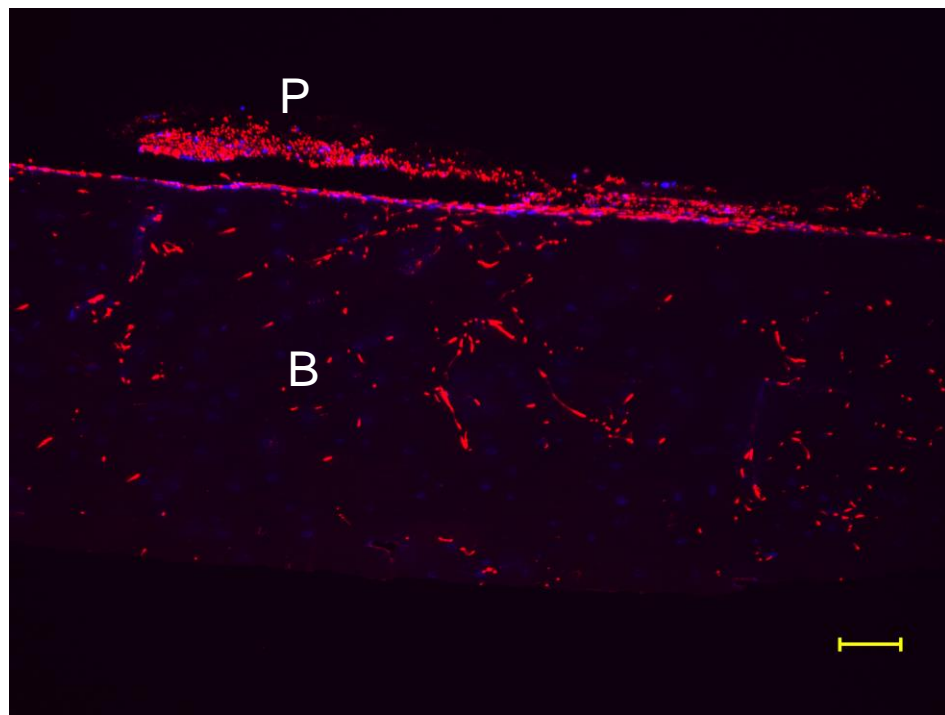

b

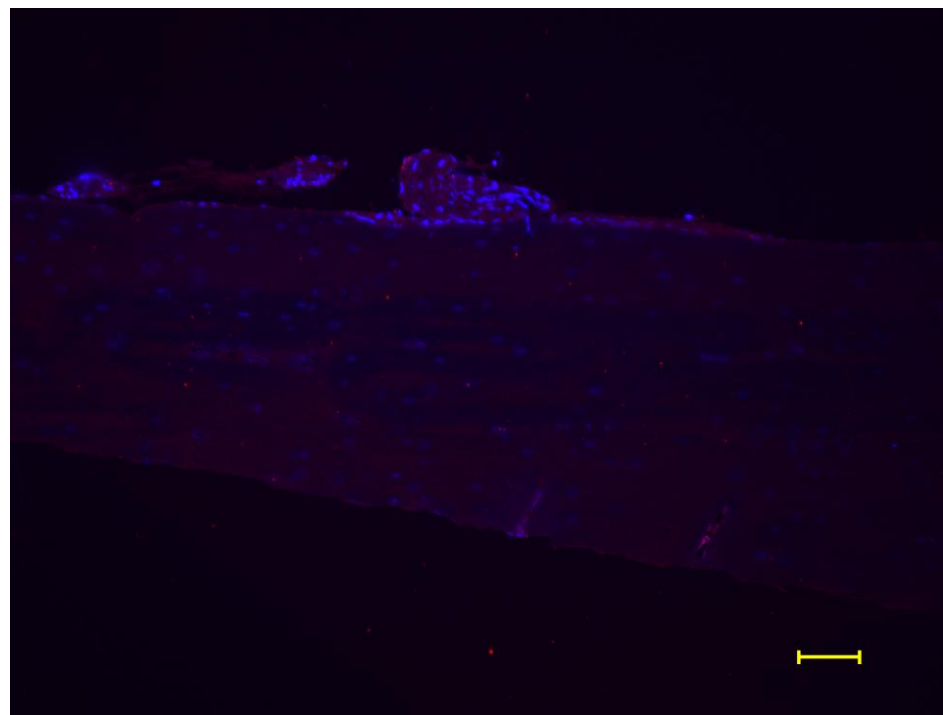

Figure S2a, b

c

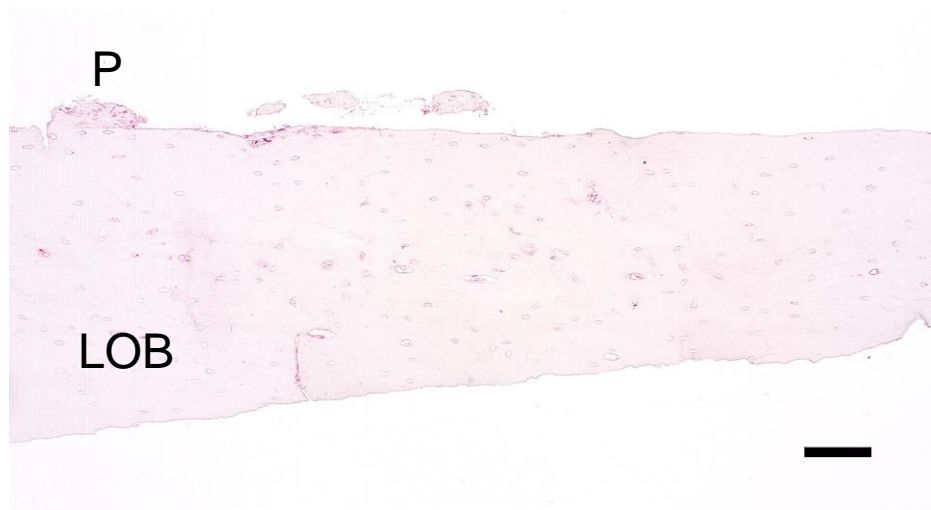

d

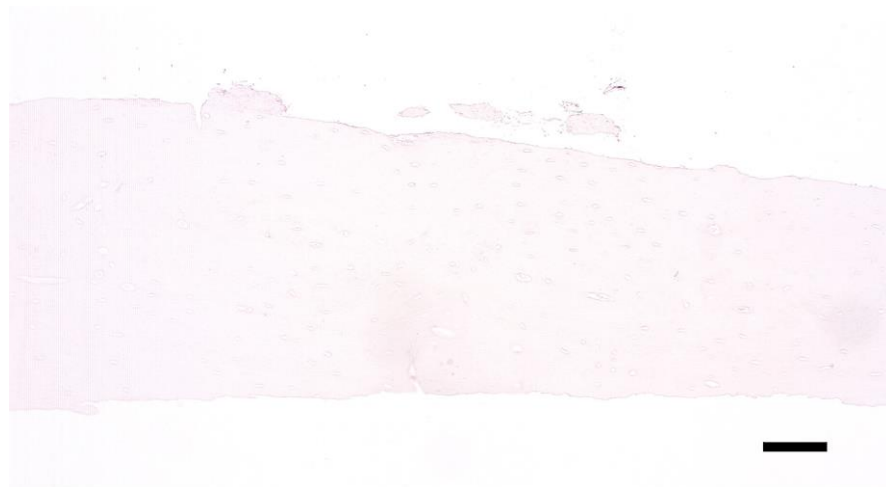

Figure S2c,d

e

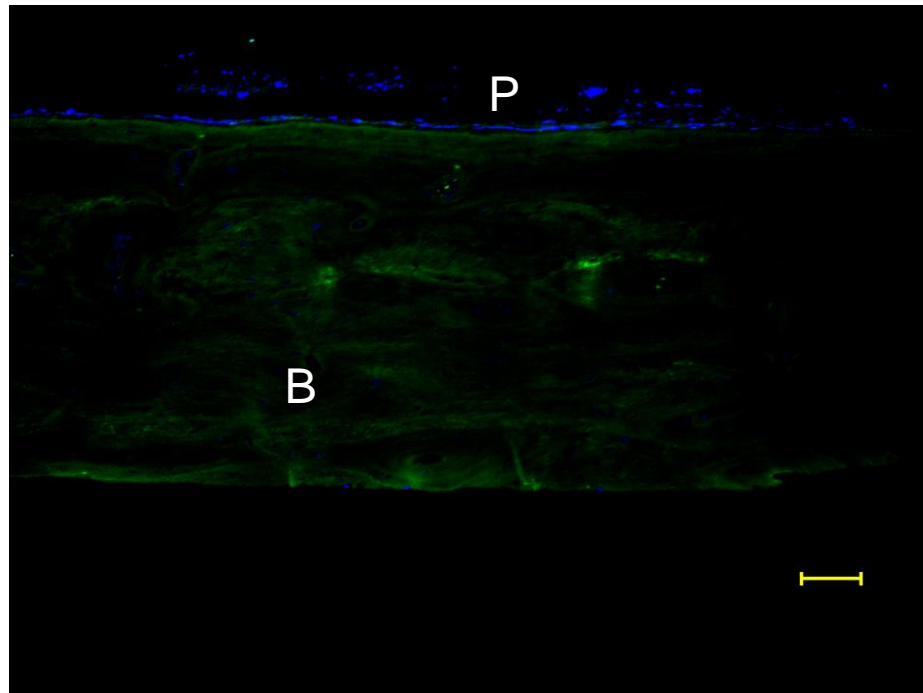

f

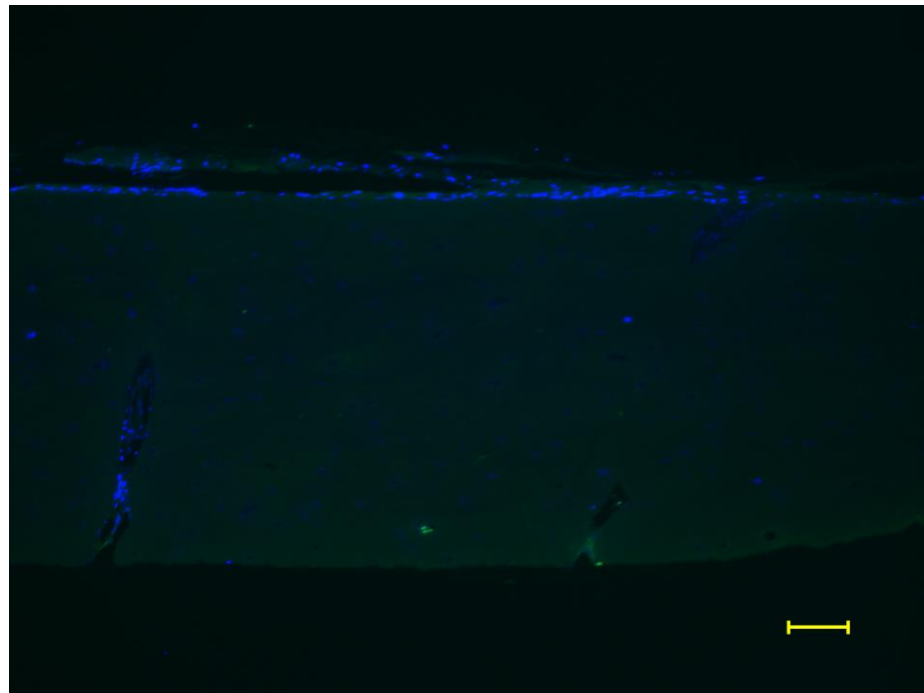

Figure S2e,f

Supplement: Supplementary file 2 — Additional file 2: Fig. S2. Fluorescent immunostaining and immunohistochemistry of bone at day 0. FBXW2 is expressed in the cambium layer, and osteocalcin is expressed in bone. Scale bar: 100 μm. (a) FBXW2: red. (b) Negative control for (a). (c) Osteocalcin. (d) RANKL: negative control for (c). (e) Osteocalcin: green. (f) RANKL: negative control for (e). P: periosteum; B: bone; LOB: lacuna of bone. [file 13104_2021_5825_MOESM2_ESM.pdf]

## Slide 1
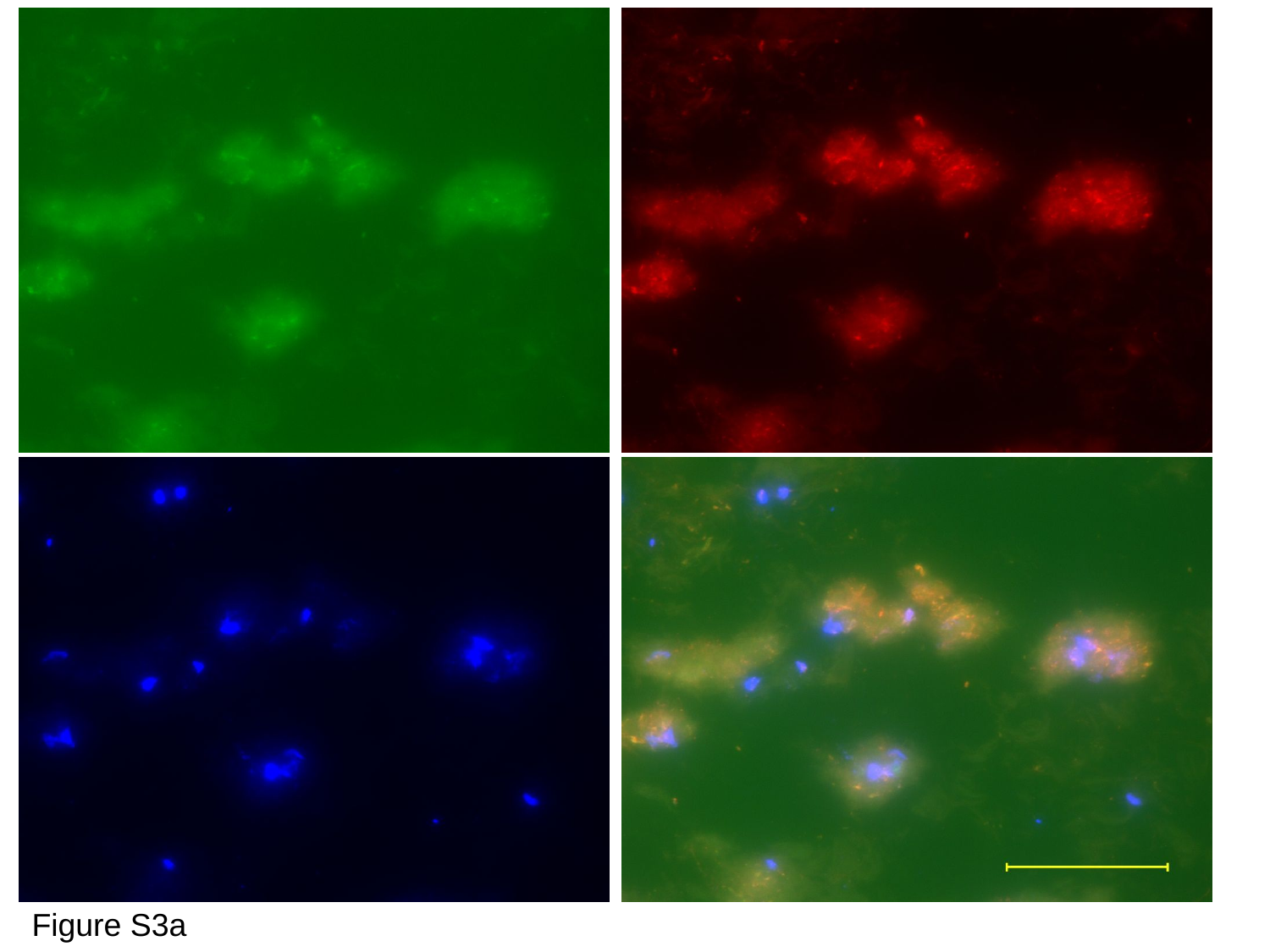

Figure S3a

## Slide 2
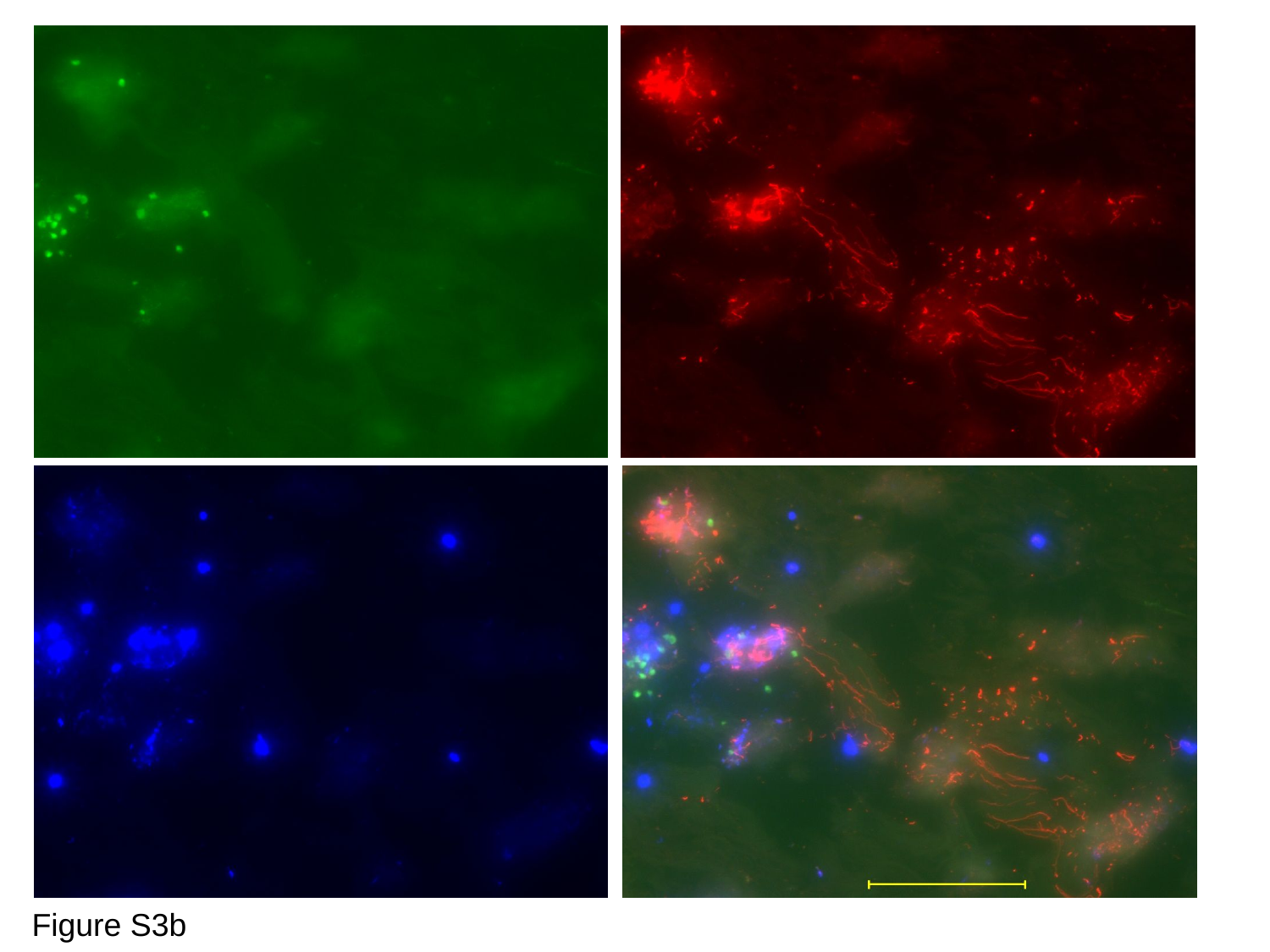

Figure S3b

Supplement: Supplementary file 3 — Additional file 3: Fig. S3. Synthesis of FBXW2. Double-fluorescent immunostaining of the periosteum at day 3 showing periosteal cell synthesis of FBXW2 and tube-like structures of FBXW2 bursting out of the cells. Scale bar: 100 μm. Osteocalcin: green; FBXW2: red; DAPI: blue. [file 13104_2021_5825_MOESM3_ESM.pptx]
