# Supplementary material for: Role of FBXW2 in explant cultures of bovine periosteum-derived cells
Source: BMC Res Notes. 2021 Nov 4;14:410. doi: 10.1186/s13104-021-05825-z (PMC8569954; doi:10.1186/s13104-021-05825-z)
Supplement: Supplementary file 4 — Additional file 4: Table S1. Changes in the expression of FBXW2 and osteocalcin. [file 13104_2021_5825_MOESM4_ESM.docx]

**Additional file 4: Table S1.** Changes in the expression of FBXW2 and osteocalcin.

Day 0 Periosteum---scarce FBXW2, osteocalcin

Cambium layer---FBXW2 expression

Bone---Osteocalcin expression, little FBXW2 expression

Week 1 Periosteum---Partial FBXW2 and scare osteocalcin expression

Week 2 Periosteum---Layer of FBXW2

Tube-like structure of FBXW2, osteocalcin

Weeks 3–7 Periosteum---appearance of periosteum-derived cells

(with FBXW2 degradation)
